# Heterogeneous Response to Immunotherapy in a Patient with Tonsillar Squamous Cell Carcinoma Assessed by ^18^F-FDG PET/CT

**DOI:** 10.3390/diagnostics11020348

**Published:** 2021-02-19

**Authors:** Artor Niccoli Asabella, Anna Giulia Nappi, Orsola Trani, Angela Sardaro, Giuseppe Rubini

**Affiliations:** 1 Nuclear Medicine Unit, AOU Policlinic “A. Perrino”, 72100 Brindisi, Italy; artnic@gmail.com (A.N.A.); orsola.30@libero.it (O.T.); 2 Section of Nuclear Medicine, DIM, University “Aldo Moro”, 70124 Bari, Italy; anna.giulia.nappi@gmail.com; 3 Section of Radiology and Radiation Oncology, DIM, University “Aldo Moro”, 70124 Bari, Italy; angela.sardaro@uniba.it

**Keywords:** tonsillar squamous cell carcinoma, immunotherapy, ^18^F-FDG PET/CT, multimodality imaging

## Abstract

Tonsillar carcinoma is the second most common malignancy of the head and neck region, with Squamous Cell Carcinoma (TSCC) as the most common histological type (>90%). For the advanced stage of TSCC, radiotherapy with or without platinum-based chemotherapy is the only therapeutic option. Immuno-checkpoint inhibitors (ICIs), in particular Nivolumab, considerably improves clinical management of these patients, but the response can be unpredictable. Difficulties can be encountered in evaluating response to immunotherapy, especially with morphological imaging, which can show an atypical response, such as pseudo-progression, leading to a premature discontinuation. Conversely, metabolic imaging can guide a more properly therapeutic decision. We present a case of a 71-year-old man affected by TSCC, treated with chemotherapy, radiotherapy, and Nivolumab as the last line of treatment. Pre- and post-immunotherapy ^18^F-FDG PET/CT showed an impressive response, avoiding early drug discontinuation and ensuring better management of this patient.

A 71-year-old male patient was histologically diagnosed with moderately differentiated Tonsillar Squamous Cell Carcinoma (TSCC) of the left anterior tonsillar, and immunohistochemical analysis was positive for p16 expression. Contrast-enhanced Computed Tomography (ceCT) confirmed the left tonsillar pillar mass without lymph node involvement. In December 2014, he began chemo- and radiotherapy, achieving a complete response (CR). After 5 years, left cervical adenopathy was palpable and subjected to biopsy sampling, which revealed metastases. Restaging ^18^F-FDG Positron Emission Tomography/CT (PET/CT) was performed and confirmed Disease Progression (DP) with a lymph node and also left lung involvement (T1aN1M1).

Oncologists decided to start chemotherapy with carboplatin and cetuximab until November 2019, when restaging ^18^F-FDG PET/CT documented a significant metabolic DP in all lesions, and consequently, chemotherapy was interrupted.

As a last line of treatment, endovenous immunotherapy (Nivolumab 10 mg/die) was chosen.

Nivolumab is an immune-checkpoint inhibitor (ICI) consisting of a well-tolerated humanized monoclonal IgG4 antibody targeting Programmed Cell Death Protein-1b (PD-1b) able to reactivate the antitumor immune response [1]. Early ^18^F-FDG PET/CT evaluation, performed 3 months after the start of immunotherapy, demonstrated a significant metabolic Partial Response (PR) with a dissociated response in different lesions and between metabolic and morphological coregistered evaluation. In particular, the left latero-cervical lymph node showed a metabolic PR with an impressive reduction of FDG uptake (maximum Standardized Uptake Value, SUVmax, of 18.7 compared to 43.3 of baseline) and a morphological CR on the CT-coregistered images (diameter of less than one centimeter). Conversely, the left pulmonary lesion showed a metabolic CR with a SUVmax <1 compared to 19.9 of baseline, while it was still visible on CT-coregistered images, but significantly reduced in size (morphological PR) (Figure 1) [2]. The patient is still continuing immunotherapy with stable disease (SD) and without significant adverse or toxic effects. 

Presently, immunotherapy, in particular ICIs, which reactivate the antitumor immunological response, represents a promising, effective, and well-tolerated therapeutic strategy for advanced TSCC patients after failure of previous treatments and in patients without other therapeutic options, improving their quality of life and overall survival (7.5 months versus 5.1 months in the control group) [1]. Clinical and radiological assessment (ceCT, Magnetic Resonance Imaging, MRI) are now considered the gold standard for immunotherapy response evaluation [3]. However, post-ICIs therapy images can be difficult to interpret for possible atypical patterns, such as pseudoprogression, hyperprogression, and dissociated response, leading to premature and incorrect discontinuation of biological agents. Pseudoprogression is an initial flare-up of tumor size linked to lymphocyte infiltration and therefore drug effectiveness, followed by tumor shrinkage. Dissociated response with a mixture of lesions responding and progressing is probably due to molecular heterogeneity between various subclones of tumor cells and the tumor microenvironment [4,5]. Hyperprogression is an unfavorable pattern with a very rapid tumor progression following immunotherapy [6,7]. Consequently, Response Evaluation Criteria in Solid Tumors (RECIST) can underestimate immunotherapeutic benefit, so new immunotherapy response criteria, such as the immune-related response criteria (irRC) and immune-related Response Evaluation Criteria in Solid Tumors (irRECIST), were proposed to more effectively assess immunotherapy response [8].

^18^F-FGD PET/CT could guarantee greater adequacy in predicting the response to immunotherapy, with a clear prognostic value [9,10], especially at an early stage, both in the case of stable or progressive disease or partial or complete response, or in the case of heterogeneous response. Changes in glucose metabolism occurring earlier than those in tumor size and assessed by an integrated visual and semiquantitative analysis [11], could promptly differentiate between responders, who should continue the ongoing treatment, and non-responders, who could benefit from alternative treatments. 

To obtain a standardized, reproducible, and accurate PET evaluation of therapeutic response in solid tumors, analogous RECIST criteria have been suggested for functional imaging, such as the European Organization for Research and Treatment of Cancer (EORTC) PET Criteria or the PET Response Criteria in Solid Tumors (PERCIST), based on quantitative measurements of changes in glucose metabolism [12,13]. However, the optimal interval time between ^18^F-FDG PET/CT and the last treatment is still a matter of debate [2,14]. Preliminary studies demonstrated a superior predictive value of progression-free survival of tumor response assessed by PERCIST than RECIST in solid tumors [2]. 

An extensive literature supports the use of ^18^F-FDG PET/CT and PERCIST criteria in early evaluation of response to immunotherapy and outcome prediction in patients with solid tumor, as there are known limitations of anatomic imaging and RECIST criteria for this purpose. Indeed, declines in tumor ^18^F-FDG uptake and quantitative measurement of treatment-induced changes in glucose metabolism precede the changes in size, so response to treatment, early detected in functional imaging, may not yet be morphologically seen [14]. 

In our clinical case, functional evaluation allowed a clear and precise assessment of the extent of disease and the response to therapy, guiding therapeutic decisions more properly than morphological imaging. Indeed, changes in glucose metabolism in the left pulmonary lesion, assessed as metabolic CR according to PERCIST criteria, preceded morphological CR, being the lesion still morphologically detected, despite reduced in size (morphological PR according to RECIST). Furthermore, functional imaging recognized the metabolic persistence of the left latero-cervical lymph node (metabolic PR according to PERCIST), which was underestimated on morphological images, being less than one centimeter (morphological CR according to RECIST).

As well as radiological assessment, atypical patterns also pose challenges in post-ICIs PET evaluation. Analogously, modified metabolic criteria have now been suggested for post-immunotherapy PET/CT study, such as iPERCIST, combining RECIST and PERCIST with the introduction of the “unconfirmed progressive metabolic disease” category, PERCRIT, PERCIMIT, and imPERCIST5 for melanoma patients or LYRIC for Hodgkin lymphoma [10,12]. However, further prospective studies need to validate these as standards for head and neck cancer patients.

## Figures and Tables

**Figure 1:**
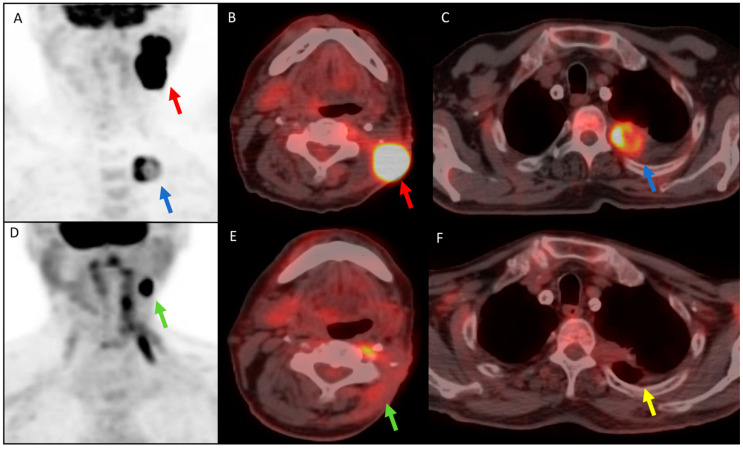
(**A**–**C**) ^18^F-FDG PET/CT performed before Nivolumab treatment. (**A**) Maximum-Intensity Projection (MIP) and (**B**,**C**) axial fused images show increased glucose metabolism in the upper left latero-cervical lymph nodes (Maximum Standardized Uptake Value, SUVmax 43.3) (red arrow), in a pulmonary lesion at the left superior lobe (SUVmax 19.1) (blue arrow), and a suspected mediastinal left lymph node at the aortopulmonary window. The disease stage was T1aN1M1. (**D**–**F**) Early ^18^F-FDG PET/CT performed 3 months after the start of immunotherapy showed a heterogeneous response in different sites and between functional and morphological co-registered evaluation: on (**D**) MIP and (**E**,**F**) axial fused images, the left latero-cervical lymph node (green arrow) impressively reduced glucose metabolism with a metabolic PR (SUVmax 18.7), while the diameter of lymph node was less than one centimeter on CT-coregistered images (morphological CR). Conversely, the left pulmonary lesion (yellow arrow) showed metabolic CR with an SUVmax <1 but a morphological PR, being still visible on CT-coregistered images although significantly reduced in size.
